# Dietary supplementation with a microencapsulated blend of organic acids and botanicals alters the kinome in the ileum and jejunum of *Gallus gallus*

**DOI:** 10.1371/journal.pone.0236950

**Published:** 2020-07-30

**Authors:** Christina L. Swaggerty, Ryan J. Arsenault, Casey Johnson, Andrea Piva, Ester Grilli

**Affiliations:** 1 U.S. Department of Agriculture, Agricultural Research Service, Southern Plains Agricultural Research Service, College Station, TX, United States of America; 2 Department of Animal and Food Sciences, University of Delaware, Newark, DE, United States of America; 3 DIMEVET, University of Bologna, Ozzano Emilia, Bologna, Italy; 4 Vetagro S.p.A., Reggio Emilia, Italy; 5 Vetagro Inc., Chicago, IL, United States of America; University of Illinois, UNITED STATES

## Abstract

The use of natural products as feed additives in the poultry industry is increasing; however, most studies focus on performance and growth with little regard for determining mechanism. Our laboratory designed a chicken (*Gallus gallus*)-specific immunometabolic kinome peptide array. Using this tool to examine the active enzymes responsible for phosphorylation events (kinases) provides important information on host and cellular functions. The objective of this project was to determine if feeding a microencapsulated product comprised of a blend of organic acids and botanicals (AviPlus^®^P) impacts the intestinal kinome of broiler chickens (*Gallus gallus*). Day-of-hatch chicks were provided 0 or 500g/MT of the additive and jejunal and ileal segments collected for kinome analysis to determine the mode-of-action of the additive. Gene ontology (GO) and Kyoto Encyclopedia of Genes and Genomes (KEGG) pathway analysis was performed by uploading the statistically significant peptides to the Search Tool for the Retrieval of Interacting Genes database. As a whole, GO and KEGG analysis showed similar activities in the ileum and jejunum. However, there were a small number of KEGG pathways that were only activated in either the ileum or jejunum, but not both. Analysis of the adipocytokine and PI3K-AKT signaling pathways showed differences between ileal and jejunal activity that were controlled, in part, by AKT3. Additionally, cytokine/chemokine evaluation showed the ileum had higher IL1β, IL6, IL10, TNFα, IFNγ, CXCL8, and CCL4 mRNA expression levels (*P*<0.05). As a whole, the data showed the addition of microencapsulated organic acids and botanicals to a broiler diet activated many of the same signaling pathways in the ileum and jejunum; however, distinctions were observed. Taken together, the findings of this study begin to define the mode-of-action that microencapsulated organic acids and botanicals have on two important intestinal segments responsible for nutrient digestion and absorption in chickens.

## Introduction

In the past 10–15 years, the poultry industry has curtailed the use of antibiotic growth promoters (AGP) in poultry production [1] and, as such, the pursuit to find suitable alternatives to enhance performance and improve animal health has grown rapidly. By the year 2025, the animal feed additive market is projected to surpass 31B USD with poultry being the largest sector based on consumption [2]. Some examples of products utilized as feed additives for poultry include: organic acids, essential oils, minerals, plant metabolites, amino acids, medicinal herbs, oligosaccharides, and various food industry and natural by-products [3–7]. For the most part, feed additive studies focus on performance or animal health benefits with little regard for how the additive is working in the host leaving a gap in the scientific knowledge.

Analysis of the host kinome with a peptide array provides site-specific protein details, has analogous biochemical properties to full-length protein, and provides a detailed picture of phosphorylation-mediated events [8] making it a powerful instrument to define mechanism(s). Phosphorylation is the foremost means of post-translational protein modification and plays a key role in almost all cell signaling events and regulation of basic biological processes [9, 10]. Peptides representing the function altering targets of kinase enzymes provide a way to characterize kinome activity [11]. Chicken-specific kinome arrays have been developed [12, 13] and are available to analyze global kinase activity providing insight on activity, phosphorylation patterns, substrate specificity, and mutational status of a specific peptide [10]. Additionally, kinome arrays can be used to identify specific immunological or metabolic pathways that are activated (or deactivated); therefore, making it possible to characterize the mode-of-action associated with a feed additive [14].

Commercial meat-type chickens consume large amounts of feed and the distinct sections of the digestive tract including the crop, proventriculus, gizzard, small intestine and the ceca all carry out specific functions [15] as the feed is turned into energy for growth. Most digestion and virtually all nutrient absorption occur in the small intestine which is made up of three segments: the duodenum, jejunum, and ileum. The increased pH of the duodenum allows the digestion process to begin followed by major nutrient digestion and absorption in the jejunum, and finally water and mineral absorption in the ileum. Microencapsulating the components of the feed additive within a lipid matrix is one approach to afford protection from the harsh environment of the digestive tract, increase stability and allow for a slow and targeted release [16, 17]. The objective of this study was to determine the kinome profile of ileal and jejunal segments isolated from broiler chickens fed a diet supplemented with a microencapsulated blend of organic acids and botanicals compared to segments from control-fed birds. We showed giving broilers a diet supplemented with the microencapsulated blend of organic acids and botanicals resulted in a distinct kinome profile that produced differences in key signaling pathways between the ileum and jejunum thus beginning to determine the mechanism(s) behind these important feed additives.

## Materials and methods

### Experimental design and chickens

Day-of-hatch by-product breeder chicks were obtained from a commercial hatchery (Timpson, TX). The chicks were transported in standard chick boxes and placed in a BL2 building in floor pens (3 m × 3 m) containing wood shavings and provided supplemental heat and *ad libitum* access to food supplied in hanging feeders and fresh water through nipple drinkers. Chickens were provided 24 hr of continual light at placement to ensure sufficient water and food intake, then transitioned to 18 hr of light and 6 hr of darkness for the remainder of the study. The temperature of the pens was maintained at 35°C for day 1 to 3, 32 to 34°C for day 4 to 7, and 29 to 31°C for day 8 to 15. Chickens were monitored each morning (08:00) for mortality, behavioral changes, litter quality, and feed and waterers were checked to ensure they were in proper working order. No mortality, behavioral changes, or other animal welfare concerns were observed during the study. The chicks were not treated with any medications or other therapeutic interventions during the study. The experiments were conducted on two separate occasions with chicks from a different hatch. Chicks were randomly assigned to either a control or supplement-fed pen (n = 20). Those assigned to the control pen were allowed *ad libitum* access to a balanced, unmedicated corn and soybean meal-based starter diet that met or exceeded the established nutrient requirements [18]. Chicks assigned to the supplement-fed pen were given free access to the same starter diet mixed with 500 g/metric ton (MT) of a microencapsulated blend of citric (25%) and sorbic (16.7%) acids, thymol (1.7%), and vanillin (1.0%) (AviPlus^®^P, Vetagro S.p.A., Reggio Emilia, Italy). The experiments were conducted in accordance with the recommended code of practice for the care and handling of poultry and followed the ethical principles according to the *Guide for the Care and Use of Laboratory Animals* [19]. All bird studies were overseen by the on-site attending veterinarian under the approved experimental procedures outlined in protocol #2017008 and were approved by the USDA/ARS Institutional Animal Care and Use Committee. The IACUC operates under the Animal and Plant Health Inspection Service (APHIS) establishment number 334299.

For both experimental replicates, the chickens on the control (n = 10) and supplemented diet (n = 10) were euthanized by cervical dislocation and necropsied at 15-days-of-age. Sampling time was established based on previous work [20, 21] and in consideration of the productive cycle of broilers. In commercial settings, most diet changes going from the starter to grower occurs between 10 and 15 days-of-age. The first two weeks are very critical to the development of the gastrointestinal and immunological function and by 2 to 3-wk-of-age broilers are considered mature and have a diversified microflora. A total of 20 chickens per treatment were used in all of the experimental procedures. A piece of jejunum (100 mg) and ileum (100 mg) was collected and rinsed with PBS to remove intestinal content, immediately flash frozen in liquid nitrogen to preserve kinase enzymatic activity, and then transferred to −80°C until further analysis using the array. In relation to Meckel’s diverticulum, the jejunum sample was collected approximately 10 cm proximal and the ileum sample was collected approximately 10 cm distally. Additional tissue samples were collected from the same areas, rinsed, and placed in RNA*later* (Qiagen, Valencia, CA), and stored at -20°C for quantitative real-time RT-PCR (qRT-PCR).

### Kinome array

Peptide array protocol was carried out as previously [14] described and summarized below using PepStar peptide microarrays from JPT Peptide Technologies GmbH (Berlin, Germany). Ileum and jejunum tissue samples were weighed and a 40 mg sample was homogenized by a Bead Ruptor homogenizer (Omni, Kennesaw GA) in 100 μL of lysis buffer (20 mM Tris–HCl pH 7.5, 150 mM NaCl, 1 mM Ethylenediaminetetraacetic acid (EDTA), 1 mM ethylene glycol tetraacetic acid (EGTA), 1% Triton X-100, 2.5 mM sodium pyrophosphate, 1 mM Na_3_VO_4_, 1 mM NaF, 1 μg/mL leupeptin, 1 g/mL aprotinin and 1 mM Phenylmethylsulphonyl fluoride). All chemicals were purchased from Sigma-Aldrich, Co. (St. Louis, MO) unless specified otherwise. Arrays were then imaged using a Tecan PowerScanner microarray scanner (Tecan Systems, San Jose, CA, USA) at 532–560 nm with a 580 nm filter to detect dye fluorescence.

### Isolation of total RNA for qRT-PCR

The tissue samples were homogenized with a BeadBug homogenizer (Benchmark Scientific, Edison, NJ). Briefly, a piece of ileum and jejunum (30–40 mg) was removed from RNA*later* and transferred to prefilled microtubes containing zirconium beads (1.5 mm; TriplePure M-Bio Grade; Benchmark Scientific). Lysis buffer (350 μL; RNeasy Mini Kit; Qiagen) was added, and the tissues homogenized for two minutes at the maximum speed. Each tissue was handled separately. Following homogenization, total RNA was isolated according to the instructions, eluted with 50 μL RNase-free water, and stored at -80°C.

### Quantitative real-time RT-PCR

Ileum and jejunum segments from control- and supplement-fed chickens were subjected to qRT-PCR to determine mRNA expression of select cytokines and chemokines as described previously [22] using published probes and primer sets [22–25]. Amplification and detection were carried out on the StepOnePlus Real-Time PCR System using Taqman RNA-to-C_T_ 1 Step Kit (Applied Biosystems, Foster City, CA). Sample standardization was done using 28S RNA. Results were calculated as 40-cycle threshold (C_T_) for each tissue sample from control- and supplement-fed chickens and the data are presented as the fold-change from controls. Fold change was calculated as 2^(supplement-fed corrected mean–control-fed corrected mean) for each tissue.

### Data and statistical analysis

Array images were then gridded using GenePix Pro software (Molecular Devices, San Jose, CA, USA), and the spot intensity signal was collected, thus ensuring peptide spots were correctly associated with their phosphorylation sites. Greater intensity fluorescence correlates to greater phosphorylation at the target site. Fluorescent intensities for treatments were then compared with controls using a data normalization program—Platform for Intelligent, Integrated Kinome Analysis, version 2 (PIIKA2) [26]. The resulting data output was then used in downstream applications such as Search Tool for the Retrieval of Interacting Genes/Proteins (STRING) and Kyoto Encyclopedia of Genes and Genomes (KEGG) databases used to pinpoint changes in protein–protein interactions and signal transduction pathways as previously described [27].

Normalization and analysis of the data was performed as previously described [28]. Images were gridded using GenePix Pro software (Molecular Devices, San Jose, CA), and the spot intensity signal was collected as the mean of pixel intensity using local feature background intensity calculation with scanner 50% gain level. The data was then analyzed using the Platform for Intelligent Integrated Kinome Analysis (PIIKA2) peptide array analysis software as previously described [26]. Briefly, the resulting data points were normalized to eliminate variation resulting from technical handling due to random variation in staining intensity between arrays or between blocks within an array. Variance stabilization and normalization was performed. Each peptide is printed in replicates of nine on the array, so there are nine values for each peptide. Using the normalized data set comparisons between treatment and control groups were performed, calculating fold-change and a significance *P*-value. The *P* value is calculated by conducting a one-sided paired *t*-test between treatment and control values for a given peptide. This consistent analysis method facilitates a more direct comparison between the two distinct array datasets and allows for a statistically robust analysis of the phosphorylation events being measured. Gene ontology (GO) and Kyoto Encyclopedia of Genes and Genomes (KEGG) pathway analysis was performed by uploading the statistically significant peptide lists to the Search Tool for the Retrieval of Interacting Genes database as previously described [29].

## Results

### Gene Ontology (GO) Biological Processes (BP)

The GO Consortium recognizes three areas relating to gene function and include cellular component, molecular function, and biological process (BP). The BP terms generally focus on the “larger processes or biological programs” that result from numerous molecular activities. Using STRING functionality, GO BP terms were generated for each tissue. The total number of BP terms associated with the ileum and jejunum were 309 and 355, respectively. Fifteen of the most significant BP terms (based on the observed gene count) for the ileum and jejunum are shown in Tables 1 and 2, respectively. The pathways in bold font are in the top 15 for each tissue. For the most part, the top 15 terms are the same in both tissues and include: cellular macromolecule metabolic process, cellular metabolic process, cellular process, cellular response to stimulus, nitrogen compound metabolic process, organic substance metabolic process, primary metabolic process, regulation of cellular process, response to stimuli, and signal transduction. The false discovery rate (FDR) for each term was highly significant for both tissues (*P* ≤ 2 × 10^−8^).

**Table 1:** GO biological processes in the ileum.

Term ID	Term Description	Gene Count	FDR*
GO:0065007	Biological regulation	47	1.6E-13
**GO:0044260**	**Cellular macromolecule metabolic process**	**34**	**1.6E-11**
**GO:0044237**	**Cellular metabolic process**	**37**	**6.7E-10**
**GO:0009987**	**Cellular process**	**50**	**5.6E-11**
**GO:0051716**	**Cellular response to stimulus**	**35**	**5.1E-15**
GO:0043170	Macromolecule metabolic process	35	1.0E-10
**GO:0006807**	**Nitrogen compound metabolic process**	**37**	**1.3E-10**
**GO:0071704**	**Organic substance metabolic process**	**38**	**2.8E-10**
GO:1901564	Organonitrogen compound metabolic process	29	4.2E-11
GO:0048518	Positive regulation of biological process	30	7.1E-13
**GO:0048522**	**Positive regulation of cellular process**	**29**	**4.2E-13**
**GO:0044238**	**Primary metabolic process**	**38**	**1.1E-10**
**GO:0050794**	**Regulation of cellular process**	**46**	**1.2E-14**
**GO:0050896**	**Response to stimulus**	**41**	**2.1E-16**
**GO:0007165**	**Signal transduction**	**31**	**9.4E-16**

* False discovery rate.

Pathways in bold are in the top 15 for the ileum and jejunum.

**Table 2:** GO biological processes in the jejunum.

Term ID	Term Description	Gene Count	FDR*
**GO:0044260**	**Cellular macromolecule metabolic process**	**26**	**2.3E-08**
**GO:0044237**	**Cellular metabolic process**	**30**	**4.6E-08**
**GO:0009987**	**Cellular process**	**41**	**4.0E-09**
**GO:0051716**	**Cellular response to stimulus**	**30**	**3.1E-13**
**GO:0006807**	**Nitrogen compound metabolic process**	**29**	**5.0E-08**
**GO:0071704**	**Organic substance metabolic process**	**30**	**6.7E-08**
**GO:0048522**	**Positive regulation of cellular process**	**27**	**3.1E-13**
**GO:0044238**	**Primary metabolic process**	**30**	**3.2E-08**
GO:0031323	Regulation of cellular metabolic process	28	1.2E-10
**GO:0050794**	**Regulation of cellular process**	**39**	**7.6E-13**
GO:0060255	Regulation of macromolecule metabolic process	27	5.6E-10
GO:0051171	Regulation of nitrogen compound metabolic process	27	3.0E-10
GO:0080090	Regulation of primary metabolic process	27	4.1E-10
**GO:0050896**	**Response to stimulus**	**34**	**1.7E-13**
**GO:0007165**	**Signal transduction**	**27**	**6.0E-14**

* False discovery rate.

Pathways in bold are in the top 15 for the jejunum and ileum.

### Kyoto Encyclopedia of Genes and Genomes (KEGG) pathways

KEGG pathways for each tissue were generated using the STRING database. For the ileum and jejunum tissues, the results were corrected using the corresponding tissue from birds on the control diet to confirm the phosphorylation changes were the result of dietary supplementation with the microencapsulated blend of organic acids and botanicals. In total, 148 and 144 KEGG pathways were significantly different in the ileum and jejunum, respectively.

As seen with the GO BP terms, most of the top 15 KEGG pathways were similar between the ileum (Table 3) and jejunum (Table 4) and were also highly significant (FDR < 1 × 10^−17^). The common pathways include: B cell receptor signaling pathway, Chemokine signaling pathway, ErbB signaling pathway, Fc epsilon RI signaling pathway, Focal adhesion, FoxO signaling pathway, HIF-1 signaling pathway, Insulin signaling pathway, MAPK signaling pathway, mTOR signaling pathway, Neurotrophin signaling pathway, and the PI3K-Akt signaling pathway and are all shown in bold font. To be included, a pathway had to be significant for each tissue over both replicate studies (n = 20 chicks/group). The pathways specific to cancer and viral infections were excluded from the analyses.

**Table 3:** Top 15 KEGG signaling pathways in the ileum.

Term ID	Pathway Description	Gene Count	FDR
4920	Adipocytokine signaling pathway	23	3.5E-22
4152	AMPK signaling pathway	34	2.1E-29
**4662**	**B cell receptor signaling pathway**	**23**	**7.4E-22**
**4062**	**Chemokine signaling pathway**	**32**	**2.9E-21**
**4012**	**ErbB signaling pathway**	**31**	**7.6E-31**
**4664**	**Fc epsilon RI signaling pathway**	**20**	**3.1E-18**
4666	Fc gamma R-mediated phagocytosis	22	3.7E-18
**4510**	**Focal adhesion**	**36**	**1.2E-23**
**4068**	**FoxO signaling pathway**	**29**	**5.4E-23**
**4066**	**HIF-1 signaling pathway**	**30**	**2.7E-26**
**4910**	**Insulin signaling pathway**	**39**	**3.8E-34**
**4010**	**MAPK signaling pathway**	**53**	**6.6E-38**
**4150**	**mTOR signaling pathway**	**20**	**2.4E-19**
**4722**	**Neurotrophin signaling pathway**	**32**	**2.8E-27**
**4151**	**PI3K-Akt signaling pathway**	**55**	**6.2E-34**

Pathways in bold are in the top 15 for the ileum and jejunum. Pathways related to cancer and viral infections were removed.

**Table 4:** Top 15 KEGG signaling pathways in the jejunum.

Term ID	Pathway Description	Gene Count	FDR
**4662**	**B cell receptor signaling pathway**	**21**	**4.8E-22**
**4062**	**Chemokine signaling pathway**	**30**	**3.2E-23**
**4012**	**ErbB signaling pathway**	**31**	**4.3E-35**
**4664**	**Fc epsilon RI signaling pathway**	**19**	**1.5E-19**
**4510**	**Focal adhesion**	**35**	**1.7E-27**
**4068**	**FoxO signaling pathway**	**23**	**2.5E-19**
**4066**	**HIF-1 signaling pathway**	**22**	**1.6E-19**
**4910**	**Insulin signaling pathway**	**33**	**2.3E-31**
**4010**	**MAPK signaling pathway**	**48**	**2.4E-39**
**4150**	**mTOR signaling pathway**	**17**	**1.2E-17**
**4722**	**Neurotrophin signaling pathway**	**32**	**6.8E-32**
**4151**	**PI3K-Akt signaling pathway**	**49**	**9.2E-35**
4015	Rap1 signaling pathway	28	2.2E-19
4014	Ras signaling pathway	40	3.7E-32
4810	Regulation of actin cytoskeleton	27	4.2E-18

Pathways in bold are in the top 15 for the jejunum and ileum. Pathways related to cancer and viral infections were removed.

There were several pathways that were completely unique to each tissue and are shown in Table 5. Of the 148 KEGG pathways in the ileal tissues, the following signaling pathways were unique and not observed in the jejunum: 2-Oxocarboxylic acid metabolism, African trypanosomiasis, Butirosin and neomycin biosynthesis, Glycerolipid metabolism, Glyoxylate and dicarboxylate metabolism, Hematopoietic cell lineage, Vasopressin-regulated water reabsorption, and Vibrio cholerae infection. The KEGG pathways that were unique to the jejunum were: Endocrine and other factor-regulated calcium reabsorption, Hedgehog signaling pathway, Ovarian steroidogenesis, and Retrograde endocannabinoid signaling. Each of these pathways were significantly (*P* < 0.04) different in the respective tissue from supplement-fed chickens compared to the comparable tissue from those on the control diet.

**Table 5:** Unique KEGG pathways observed in the ileum or jejunum, but not both tissues, from supplement-fed chickens compared to those on the control diet.

Ileum				Jejunum			
Term ID	Pathway Description	Gene Count	FDR*	Term ID	Pathway Description	Gene Count	FDR
1210	2-Oxocarboxylic acid metabolism	3	0.007	4961	Endocrine and other factor-regulated calcium reabsorption	3	0.045
5143	African trypanosomiasis	3	0.042	4340	Hedgehog signaling pathway	4	0.009
524	Butirosin and neomycin biosynthesis	3	0.0001	4913	Ovarian steroidogenesis	5	0.001
561	Glycerolipid metabolism	5	0.007	4723	Retrograde endocannabinoid signaling	5	0.018
630	Glyoxylate and dicarboxylate metabolism	3	0.022				
4640	Hematopoietic cell lineage	5	0.042				
4962	Vasopressin-regulated water reabsorption	4	0.018				
5110	Vibrio cholera infection	4	0.029				

* False Discovery Rate.

### Adipocytokine and PI3K-AKT signaling pathways

The adipocytokine signaling pathway was one of the top 15 pathways in the ileum (Table 3) but was not in the top 15 pathways of the jejunum (Table 4). The adipocytokine signaling pathway is involved in several other key pathways including insulin signaling, MAPK signaling, TNF signaling, and NF-κB signaling pathways, all of which were impacted by the microencapsulated blend of organic acids and botanicals used in this study. As expected, further evaluation of this pathway showed specific proteins were significantly different in the ileum and jejunum (Table 6). The ileum-specific proteins included: AKT3, CAMKK1, CAMKK2, CPT1A, PPARA, PPARGC1A, PRKAB1, PRKAG2, STK11, and TRAF2. The jejunum-specific proteins were: MAPK8, PRKAA2, and PRKCQ.

**Table 6:** Individual proteins in the Adipocytokine and PI3K-AKT signaling pathways that are significantly different in tissues from supplement-fed chickens compared to controls.

Adipocytokine signaling pathway		PI3K-AKT signaling pathway			
Ileum	Jejunum	Ileum		Jejunum	
ACACB ACSL4 ACSL5 ACSL6 **AKT3** **CAMKK1** **CAMKK2** CHUK **CPT1A** IRS1 JAK2 MTOR NFKB1 **PPARA** **PPARGC1A** PRKAA1 **PRKAB1** PRKAB2 **PRKAG2** SOCS3 STAT3 **STK11** **TRAF2**	ACACB ACSL4 ACSL5 ACSL6 CHUK IRS1 JAK2 **MAPK8** MTOR NFKB1 PRKAA1 **PRKAA2** PRKAB2 **PRKCQ** SOCS3 STAT3	**AKT3** ATF2 CCND1 CCNE1 **CDK6** CHUK **CREB1** CSF1R EGFR EIF4EBP1 FGF20 FGFR1 FGFR2 FGFR3 FGFR4 **FLT4** GRB2 GSK3B HSP90AB1 **HSP90B1** IFNAR1 IL7R IRS1 **ITGA6** **JAK1** JAK2 KDR KIT	MAP2K1 **MAP2K2** MAPK1 MET MLST8 MTOR NFKB1 PDGFRA PDGFRB PDPK1 PIK3CB PIK3CG PPP2CA PPP2R5D PRKAA1 PRKCA PTEN PTK2 RPS6KB1 **RPTOR** **SOS1** **STK11** SYK THBS1 **THEM4** TSC2 YWHAZ	ATF2 CCND1 CCNE1 CHUK CSF1R EGFR EIF4EBP1 FGF20 FGFR1 FGFR3 FGFR4 GRB2 GSK3B **HRAS** HSP90AB1 IFNAR1 IL7R **INSR** IRS1 JAK2 KDR KIT	MAP2K1 MAPK1 MET MLST8 MTOR NFKB1 **NGFR** PDGFRA PDGFRB PDPK1 **PIK3AP1** PIK3CB PIK3CG PPP2CA PPP2R5D PRKAA1 **PRKAA2** PRKCA PTEN PTK2 **RAC1** **RAF1** RPS6KB1 SYK THBS3 TSC2 YWHAB

Proteins in bold type are unique to that tissue.

Pathways that were top 15 in ileum but not jejunum included adipocytokine signaling pathway, AMPK signaling pathway and Fc gamma R-mediated phagocytosis, all of these contain the central signaling molecule AKT. In addition, the phosphatidylinositol 3-kinase-AKT (PI3K-AKT) signaling pathway was highly significant (FDR < 6 × 10^−34^) in both tissues, but not all of the same proteins were represented in the two tissues (Table 6). The ileum-specific significant proteins included: AKT3, CDK6, CREB1, FLT4, HSP90B1, ITGA6, JAK1, MAP2K2, RPTOR, SOS1, STK11, and THEM4 while the jejunum-specific proteins were HRAS, INSR, NGFR, PIK3AP1, PRKAA2, RAC1, and RAF1. When considering the adipocytokine and PI3K-AKT signaling pathways, AKT3 (RAC-gamma serine/threonine-protein kinase) was the only common protein which was observed in the ileum while not present in the jejunum when comparing supplement-fed birds to controls. Further, AKT3 is known to impact both metabolic and immune pathway activation.

### Clustering of KEGG pathways

**Fig 1:**
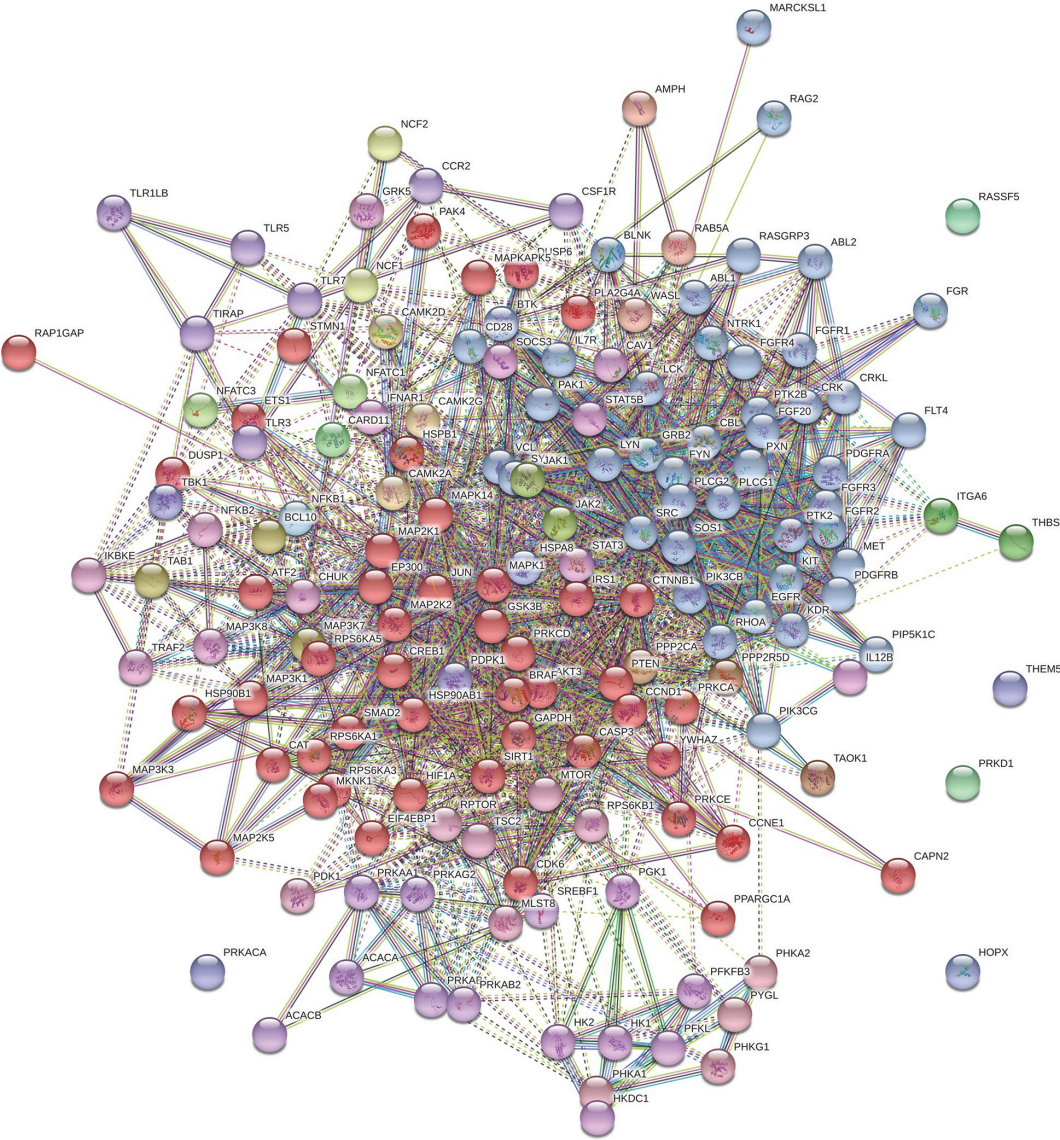
Markov Cluster of significant proteins in the ileum. Ileal protein-protein interactions in ileum were analyzed using the Markov Cluster (MCL) algorithm (*P* ≤ 0.05) in the STRING database.

**Fig 2:**
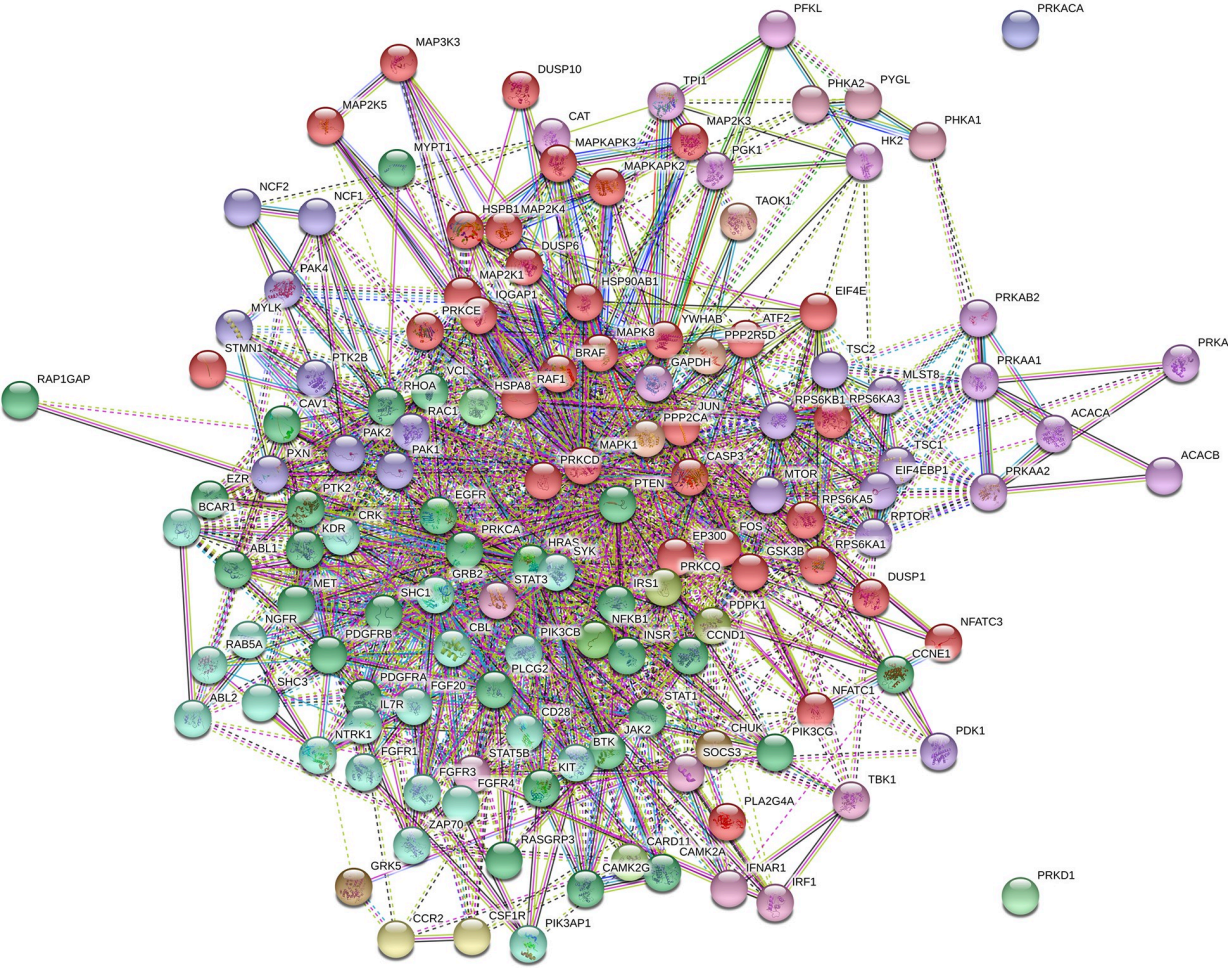
Markov Cluster of significant proteins in the jejunum. Jejunal protein-protein interactions in jejunum were analyzed using the Markov Cluster (MCL) algorithm (*P* ≤ 0.05) in the STRING database.

The protein-protein interactions of all statistically (*P* ≤ 0.05) different proteins in the ileum and jejunum were subjected to the Markov Cluster (MCL) algorithm. Using the STRING database MCL clustering tool, the clusters were generated and are shown for the ileum and jejunum in Figs 1 and 2, respectively. The MCL algorithm was pre-set to generate three distinct clusters. Even though the observed KEGG pathways were similar between the two tissues, the resulting diagrams clearly showed that distinct clusters emerged based on tissue type.

### Cytokine and chemokine mRNA expression

**Fig 3:**
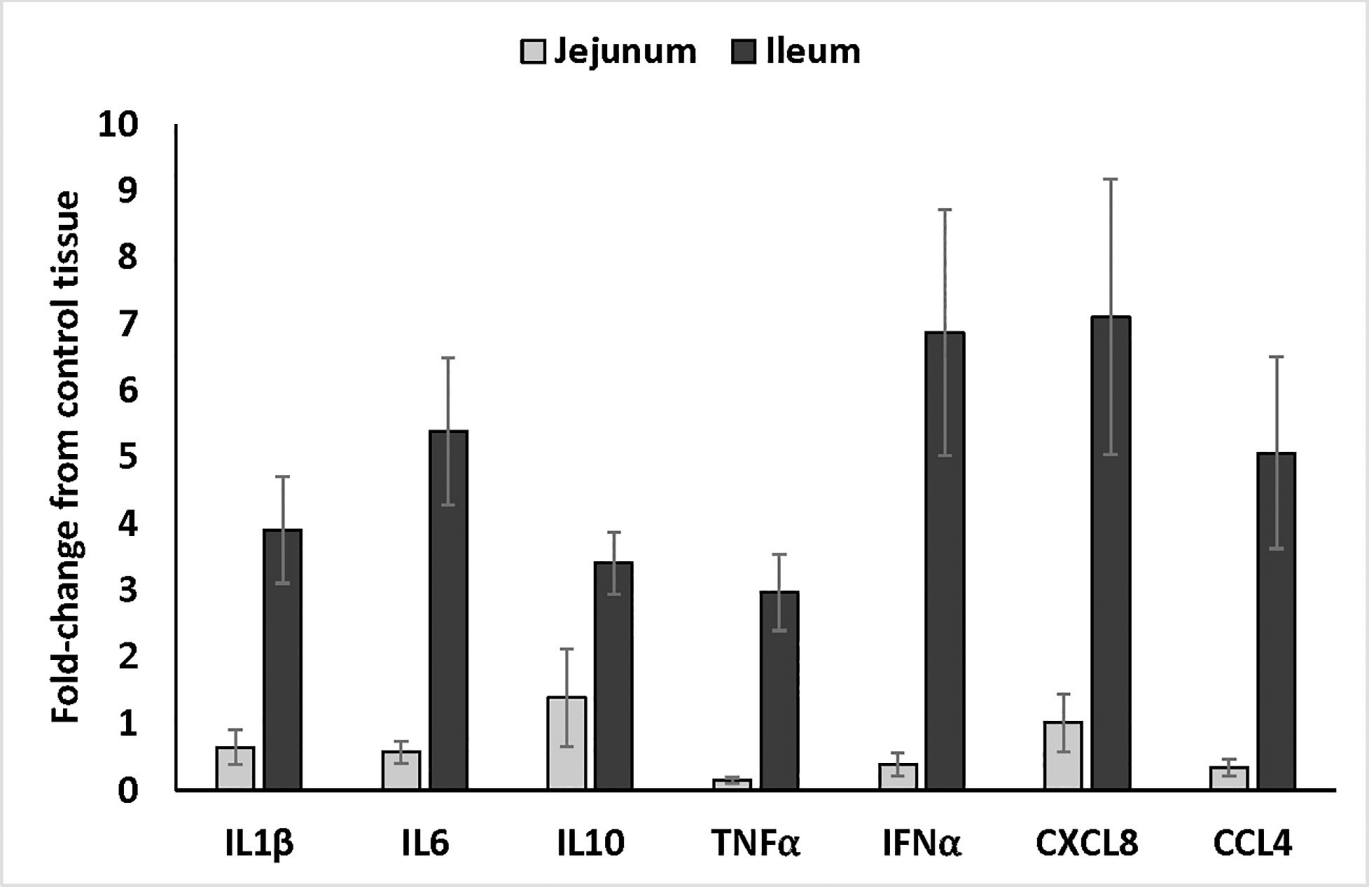
Cytokine and chemokine mRNA expression in ileum and jejunum samples from supplement-fed chickens compared to those on control diet. Quantitative real-time RT-PCR was used to validate the array data. The mRNA expression levels of various cytokines and chemokines were measured in Ileal (n = 10 treatment [5 per experiment]) and jejunal (n = 10 treatment [5 per experiment]) samples from control and supplement-fed chickens. Sample standardization was done using 28S RNA. Results were calculated as 40-cycle threshold (C_T_) for each tissue sample from control- and supplement-fed chickens and the data are presented as the fold-change from controls. Fold change was calculated as 2^(supplement-fed corrected mean–control-fed corrected mean) for each tissue. Data are presented as the fold-change comparing the supplement-fed tissue samples to the corresponding control sample.

The chemokine signaling pathway was significantly different in both tissues (Tables 3 and 4). Additionally, the differences observed between the Adipocytokine and PI3K-AKT signaling pathways (Table 6) indicated cytokine and chemokine mRNA expression could be used for validation of the array data. Therefore, the mRNA expression levels of select cytokines (IL1β, IL6, IL10, TNFα, and IFNα) and chemokines (CXCL8 and CCL4) were determined in ileum and jejunum samples collected from control- and supplement-fed chickens (Fig 3). For all cytokines and chemokines that were measured, the mRNA expression levels in the ileum from supplement-fed chickens were three to seven-fold higher (*P* < 0.05) compared to expression in the tissue from the control-fed chickens whereas the expression levels in the jejunum only changed from 0.15 to 1.4 -fold above the controls. These data showed the differences observed in the array were supported by differential activity in the form of cytokine and chemokine mRNA expression between the ileum and jejunum in supplement-fed chickens compared to controls.

## Discussion

The blend of organic acids and botanicals used in the current study has been shown to enhanced gut immune and barrier function in the ileum and jejunum of weaned pigs [20]; however, the impact and mode-of-action of these dietary organic acids and botanicals is not fully understood in poultry. Therefore, in the present study we evaluated the kinome profile in poultry using samples from the ileum and jejunum as both tissues display similarities with mammals in terms of structure and function. Kinome analysis provides a detailed picture of the phosphorylation patterns of specific peptides while highlighting the various signaling pathways that are associated with the representative proteins involved [10]. The availability of chicken-specific kinome arrays [12, 13] provides poultry researches with a powerful tool for defining mechanism and mode-of-action in experimental conditions, including those associated with feed additives [14]. The microencapsulated blend of organic acids and botanicals used in this study (AviPlus^®^ P; identification number 4d3) is recognized by the European Union Commission and European Food Safety Authority (EFSA) for its ability to enhance growth and feed efficiency in healthy chickens. However, the precise mechanism(s) driving the improved feed efficiency and growth rate has not been fully characterized. The study described herein was designed to address this gap in knowledge and function by evaluating the kinome profile of ileal and jejunal segments isolated from broiler chickens fed a diet supplemented with a microencapsulated blend of organic (citric and sorbic) acids and botanicals (thymol and vanillin) compared to segments from control-fed birds.

Use of microencapsulation allows for the slow release of active compounds, such as organic acids and botanicals, into the intestinal tract instead of disappearing after exiting the stomach [16]. Additionally, a study by Grilli and colleagues showed that microencapsulation allows for the slow release of organic acids in the small intestine of broilers [17]. A chicken kinome array was used to determine if there were major differences between key signaling pathways comparing ileum and jejunum segments in supplement-fed chickens compared to those on a control diet. As a whole, the most significant GO BP (Tables 1 and 2) and KEGG pathways (Tables 3 and 4) were similar between these two segments of small intestine. The common GO BP terms centered around cellular and macromolecule metabolic processes and the response and regulation of cellular processes. Analysis of the KEGG signaling pathways highlighted both immune- and metabolic-related pathways including FoxO, MAPK, insulin, mTOR, and chemokine signaling pathways. The results obtained herein are consistent with another kinome study looking to determine the host response to the addition of a feed additive. In that study, the authors also identified the key BP and KEGG pathways providing mechanistic information on function [14]. Taken together, these studies show the usefulness of kinome data to provide insight into mode-of-action.

The avian digestive tract includes the crop, proventriculus, gizzard, small intestine, and ceca with much focus on the crop and ceca; however, less is known about a properly functioning small intestine. Collectively, digestion and nutrient absorption occurs in the small intestine which is comprised of three segments including the duodenal loop, jejunum, and ileum. As might be expected, based on their close proximity, there are some overlapping functions between the jejunum and ileum. However, each segment does function independently with nutrient digestion and absorption mainly occurring in the jejunum with water and mineral adsorption primarily taking place in the ileum as well as starch digestion and absorption in commercial fast-growing chickens [15, 30].

Numerous studies have evaluated cytokine or chemokine expression in various tissues following dietary supplementation with a feed additive [31–33] or even in the presence of basal diets without further amendments [34]. In the study described herein, supplementation with the blend of organic acids and botanicals showed increased mRNA expression of wide-ranging cytokines and chemokines that were consistently higher in the ileum compared to the levels observed in the jejunum. These data are in agreement with a study showing probiotic supplementation upregulated IL8 (CXCL8) and IL10 in the ileum [35]. In a separate study, the addition of β-glucan as a dietary amendment produced a differential cytokine response in the small intestine including the ileum and jejunum [33]; however, the consistent trend of the ileum having higher expression levels compared to the jejunum were not observed in that study. These differences could be due, in part, to the birds used, there were also differences in some of the cytokines measured, and lastly, the distinctly different type of feed additive that was evaluated (prebiotic in β-glucan compared to organic acids and botanicals). Further, addition of cationic peptides to a broiler’s diet alters the cytokine and chemokine mRNA expression [32]. This study shows reduced expression of IL1β, IL6, and IFNγ but all of these measures are from the ceca and not the small intestine as measured in the current study. Addition of butyric acid, an organic acid, to the diet of young piglets showed varying expression levels of cytokines along different segments of the intestinal tract [36]. Collectively, all of the studies showed dietary supplementation with diverse products ranging from prebiotics, peptides, or organic acids and botanicals has the ability to impact cytokine and chemokine expression in the gastrointestinal tract.

Clearly, cytokine and chemokine responses are observed in chicken intestinal segments following supplementation with various natural compounds that are intended to replace antibiotics and/or act as growth and performance enhancers. A deeper evaluation of the kinome data showed the PI3K-AKT and adipocytokine signaling pathways were important in the tissues from the supplement-fed chickens compared to those on the control diet (Tables 3 and 4). Specifically, AKT3, a serine and threonine kinase, is one of the unique proteins that was different in the ileum while not in the jejunum (Table 6). AKT3 lies upstream of NF-κB [37–39]. NF-κB is important for an array of cellular functions including, but not limited to, immune cell maturation/differentiation and pro-inflammatory cytokine/chemokine expression [40]. In the current study, increased levels of pro-inflammatory cytokine/chemokine expression were observed (Fig 3) in the ileum and to a much lesser extent in the jejunum; therefore, it is possible that AKT3 and NF-κB activation are mediating these differences. Activation of the PI3K-AKT pathway has also shown increases in IL8 (CXCL8) and IL10 expression in vitro [41]. In addition to the immunological changes, AKT signaling directs host metabolism and increases protein synthesis [42]. Even though performance was not considered in the current study, the microencapsulated blend of organic acids and botanicals (AviPlus^®^ P; EFSA identification number 4d3) is recognized by the EU as an approved feed additive that improves feed efficiency and growth in healthy broilers. It is possible the observed differences in intestinal kinome activity contribute to the enhanced performance, but additional studies are required to draw this correlation.

In addition to AKT3, downstream phosphorylation of the transcription factor CREB1 (CAMP responsive element binding protein 1) was also unique to the ileum (Table 6) which could contribute to increased IL6 and TNFα, both of which, have the potential to affect lipid metabolism and immune function [43]. The organic acid blend used in the current study contains citric and sorbic acids. Butyrate, a short chain fatty organic acid, and its derivatives, are commonly used as feed amendments by the poultry industry [44] and has been shown to promote proliferation and maturation of intestinal cells [45] that may be mediated by CREB [46]. Organic acids make up the largest component of the blend used in the present study, and as seen with the butyrate studies, it is possible that the citric and sorbic acids are driving the signaling via CREB activation.

We have shown giving broilers a diet supplemented with the microencapsulated blend of organic acids and botanicals results in a distinct kinome profile in the ileum compared to the jejunum, and future studies will further explore these specific immune and metabolic pathways. From a purely scientific standpoint, understanding mechanism(s) is important, but even more so, this understanding will be vital for the poultry industry moving forward so they can make decisions based on sound science.

## Supporting information

S1 Data Data used in the study. (XLSX)

S2 Data Data used in the study. (XLSX)
